# Comparative behavioural observations demonstrate the ‘cleaner’ shrimp *Periclimenes yucatanicus* engages in true symbiotic cleaning interactions

**DOI:** 10.1098/rsos.170078

**Published:** 2017-04-26

**Authors:** Benjamin M. Titus, Clayton Vondriska, Marymegan Daly

**Affiliations:** Department of Evolution, Ecology, and Organismal Biology, The Ohio State University, 1315 Kinnear Road, Columbus, OH 43210, USA

**Keywords:** cleaner shrimp, cleaning behaviour, mimicry, coral reefs, anemones, symbiosis

## Abstract

Cleaner shrimps are ecologically important members of coral reef communities, but for many species, cleaner status (i.e. dedicated, facultative and mimic), clientele and ecological role remain unverified or described. On Caribbean coral reefs, the spotted ‘cleaner’ shrimp *Periclimenes yucatanicus* forms symbioses with sea anemones that may serve as cleaning stations for reef fishes. The status of this species as a cleaner is ambiguous: only a single *in situ* cleaning interaction has been reported, and in the only test of its efficacy as a cleaner, it did not effectively reduce parasite loads from surgeonfish. It has subsequently been hypothesized by other authors to be a cleaner mimic. We conduct a comparative investigation of cleaning behaviour between *P. yucatanicus* and the ecologically similar, closely related, dedicated cleaner shrimp *Ancylomenes pedersoni* in Curacao, Netherlands Antilles. We provide the first detailed field observations on cleaning behaviour for *P. yucatanicus* and test multiple behavioural expectations surrounding mimicry in cleaning symbioses. We found that *P. yucatanicus* regularly signals its availability to clean, client fishes visit regularly and the shrimp does engage in true symbiotic cleaning interactions, but these are brief and our video reflects a species that appears hesitant to engage posing clients. In comparison to *A. pedersoni*, *P. yucatanicus* stations had significantly fewer total visits and cleans, and 50% of all cleaning interactions at *P. yucatanicus* stations were shorter than 10 s in total duration. Our behavioural observations confirm that *P. yucatanicus* is a true cleaner shrimp; we reject the hypothesis of mimicry. However, investigation is needed to confirm whether this species is a dedicated or facultative cleaner. We hypothesize that *P. yucatanicus* has a specialized ecological role as a cleaner species, compared to *A. pedersoni*.

## 1. Introduction

Cleaner organisms (e.g. gobies, wrasse and shrimps) are ecologically important service providers on tropical coral reefs [1–5] that remove potentially harmful ectoparasites from reef fishes [1,2,4,5]. Cleaners can reduce client ectoparasite loads [1,2,4,5] and decrease client stress [6,7], and their mere presence affects client habitat choice [8] and community-wide biodiversity [9,10]. To date, dozens of reef organisms have been labelled ‘cleaners’, but we have scant knowledge of the cleaning activities of many of these species, and their ecological importance and clientele may be unverified or described [3].

Cleaner species are broadly categorized as either dedicated, recently defined by Vaughan *et al*. [11] as a species committed to a cleaning lifestyle for all of their non-larval ontogeny, or facultative, defined as a species committed to a cleaning lifestyle for only part of their non-larval ontogeny [3,11]. However, many species are designated as cleaners through anecdotal data, the grey literature and one-off interactions (reviewed by Vaughan *et al*. [11]). These often come with little evidence to support that these observations are not the result of poor taxonomic identification or incidental cleaning, or whether they enhance client fitness [2,3,11,12]. Defining a species as a cleaner also implies that their interactions with clients are symbiotic. Vaughan *et al*. [11] have now extended the definition of a cleaning symbiosis to include communicative behaviours (i.e. signalling) that precede cleaning interactions. These include both the signals demonstrating the willingness and availability of the cleaner, and the subsequent pose and submission of the client [11]. Thus, without systematic study, the cleaner label is likely to be overextended in many cases, misattributing the ecological role and importance of many species and larger taxonomic groups.

A total of 208 fish species and 51 shrimp species are classified as cleaners [11]. Cleaner fishes have received far more attention in the literature, although shrimps have been shown to be equally important cleaner species (reviewed by Vaughan *et al*. [11]). Furthermore, the cleaner definition appears to be more broadly applied to shrimps than to fishes, with entire families and genera incorrectly defined as cleaners [11]. The genus *Periclimenes* (Palaemonidae) has over 175 described species, all of which have been referred to as cleaner shrimps [13]. However, after the designation of the genus *Ancylomenes* [14], Vaughan *et al*. [11] note that only one species remaining in *Periclimenes* (*P. yucatanicus*) has actually been observed engaging in a cleaning interaction [15]. Interestingly, the evidence supporting the designation of *P. yucatanicus* as a true symbiotic cleaner shrimp is weak [15], and it has even been hypothesized to be a cleaner mimic [4,16], possibly using similar coloration to increase foraging ability and/or decrease predation pressure [17–23]. Thus, there is little support that any species within *Periclimenes* are dedicated or facultative cleaners.

Here, we use standardized observations of *in situ* cleaning to conduct the first field-based assessment of the cleaning behaviour and clientele of the spotted cleaner shrimp *P. yucatanicus* in Curacao, Netherlands Antilles. Additionally, we test a series of behavioural expectations (see Material and methods) surrounding mimicry in cleaning symbioses for *P. yucatanicus* to determine if previous speculation (e.g. [4,16]) surrounding mimicry in this species is warranted. We take a comparative approach by incorporating observations from a well-studied, closely related, dedicated cleaner species, Pederson's cleaner shrimp *Ancylomenes pedersoni* (e.g. [4,24]).

**Figure 1.**
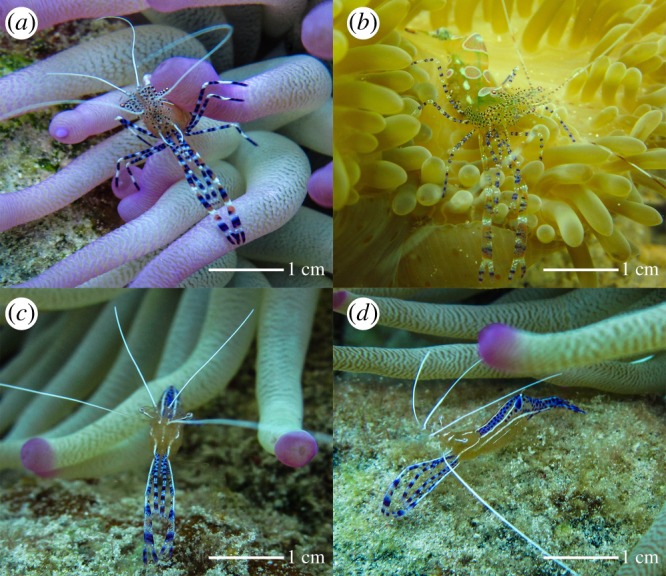
Representative images of the spotted cleaner shrimp *Periclimenes yucatanicus* (*a,b*), and Pederson's cleaner shrimp *Ancylomenes pedersoni* (*c,d*). Anemone hosts in (*a,c,d*) are *Condylactis gigantea*. Anemone host in (*b*) is *Stichodactyla helianthus*. Images are from Curacao, Netherlands Antilles (*a,c,d*), and Bocas del Toro, Panama (*b*).

The evidence that *P. yucatanicus* is a true cleaner is limited to a single *in situ* observation by Spotte *et al*. [15] from the Turks and Caicos. Only one study has been conducted on the cleaning efficacy of *P. yucatanicus*, and in this mesocosm experiment [4] it was ineffective in reducing monogenean flatworm loads on surgeonfish. However, Titus *et al*. [25] hypothesized that cleaner species may specialize on different ectoparasites, which would reduce competition with other cleaners and allow for broadly overlapping client diversity. Over 20 families of reef fishes are documented as clients of *A. pedersoni*, and the signalling behaviour, visitation rate and interaction lengths across a number of client species are well characterized [1,4,15,16,24–30]. However, none of this information is known for *P. yucatanicus*, despite both shrimp being sea anemone symbionts on Caribbean coral reefs, having similar patterns of host use (e.g. [31–33]), co-occurring on the same individual anemone host [32,34], superficially resembling each other in terms of size, pattern, coloration (figure 1) and being closely related [14,34,35].

These similarities are central to the hypothesis that *P. yucatanicus* is a mimic of *A. pedersoni*. While closely related, they are not sister taxa [14,34,35], and the classic cleaner patterns and coloration both species possess could be convergent or a shared primitive feature inherited from their common ancestor. In most localities, *A. pedersoni* is more abundant than *P. yucatanicus* [32,36], satisfying the requirement for negative frequency-dependent selection expected of protective mimicry scenarios (e.g. [37]). To date, mimicry has only been described between the juvenile bluestripe cleaner wrasse (*Labroides dimidiatus*: true cleaner) and the fangtooth blenny (*Plagiotremus rhinorhynchos*: cleaner mimic; e.g. [19,22,23,38,39]): the fangtooth blenny is an aggressive mimic, increasing foraging success by duping posing clients and then ‘attacking’, removing scales and live tissue [19,22]. No mimics have been described among cleaner shrimp.

## 2. Material and methods

### 2.1. Comparative behavioural observations

Accurately delineating the status of a species as a cleaner can be challenging. Standardized methods revolve around in-depth field observations to assess the frequency at which a prospective cleaner species is visited (proxy for ecological importance) [40], the length of the services provided (e.g. [40]), gut content analyses and food preference trials (e.g. [26,41–44]), manipulative experiments to determine the efficacy of parasite removal (e.g. [4]) and signalling and behavioural observations. Here, we focus solely on *in situ* client visitation rate and clean length and provide observations of cleaner signalling.

Comparative behavioural observations for both *P. yucatanicus* and *A. pedersoni* were conducted over 10 days between 24 May and 2 June 2015 on the same fringing reef adjacent to the Caribbean Marine Biological (CARMABI) Research Institute in Curacao, Netherlands Antilles (12°06′32.4^″^ N; 68°56′0.7^″^ W). The CARMABI house reef is approximately 50 m from shore; the reef crest is shallow (4–6 m) and quickly slopes to depths greater than 25 m. No major ecological data were quantified, but observationally the CARMABI reef is characterized by low scleractinian coral cover (qualitative estimate less than 10%) and large, crustacean-hosting sea anemones are common. Both *P. yucatanicus* and *A. pedersoni* were common at our study site when we conducted the trials and were readily discernible from one another through in-life colour patterns; elsewhere, *P. yucatanicus* is typically less abundant than *A. pedersoni* [32,36]. As on other Caribbean reefs, not all anemones hosted crustacean symbionts [32].

Before conducting behavioural observations, we measured (tentacle crown surface area (TCSA), in cm^2^; after Huebner *et al.* [45]), mapped and tagged only those anemones hosting *P. yucatanicus* (*n* = 30) and *A. pedersoni* (*n* = 15) over an approximately 80 × 20 m area of reef (approx. 1600 m^2^). All anemones used in this study were on reef crest and shallow fore reef habitats between 4 and 8 m depth. While we would have ideally controlled for anemone host, that was not feasible given the patterns of host use at this site. All *P. yucatanicus* hosted with their preferred host, the giant Caribbean anemone *Condylactis gigantea* [32], while all but one of the *A. pedersoni* stations were found in association with the corkscrew anemone *Bartholomea annulata* (one *A. pedersoni* was hosted by *C. gigantea*). While intraspecific group sizes generally do vary [31,32,36], all *P. yucatanicus* stations at our study site hosted only one shrimp. Shrimp group sizes for *A. pedersoni* stations varied between one and six shrimp, but only three of these hosted more than a single shrimp.

Each day, we deployed GoPro Hero3 video cameras at individual stations we had not previously recorded video from and exited the water following Titus *et al*. [25,30], to reduce the potential for diver presence to disrupt cleaning interactions (e.g. [30]). We could not deploy cameras at each cleaning station each day because we were limited to four video cameras. Anemones with shrimp were selected randomly following initial mapping. Owing to time constraints, we did not record every tagged station. Our final data set had *n* = 23 cleaning stations for *P. yucatanicus* and *n* = 12 stations for *A. pedersoni*. Cameras were mounted to lead dive weights and placed approximately 1 m from each anemone station. An even representation of video was recorded continuously at each station (median = 150 min; IQR: 127.5–150 min) for the duration of battery life (approx. 100–200 min), with only one station recording less than 100 min of video. The duration of each recording did not significantly correlate with client visitation at either *P. yucatanicus* (*r*^2^ = 0.02, *p* = 0.55) or *A. pedersoni* (*r*^2^ = 0.0005, *p* = 0.94) cleaning stations. Cameras were typically deployed at 8.00, but we did not control for time of day because Titus *et al*. [25] showed that time of day does not affect cleaning frequency for *A. pedersoni*. We did not video anemones lacking cleaner shrimp to control for whether anemone species differentially attract client fishes.

Videos were downloaded and cleaning interactions analysed after Huebner & Chadwick [28,29]. We quantified (i) client visitation rate (visits min^−1^), with a visit defined as a pause, or pose, directly in front of the cleaning station that lasted at least 2–3 s regardless of whether a cleaning bout immediately followed [28,46]. (ii) Clean rate (cleans min^−1^) and the length of the interaction (s), with a clean being defined as physical contact between the shrimp and posing client reef fish [25,28,29]. (iii) Client flinches, a proxy for cheating frequency, defined as a rapid flinch or jolt by the client reef fish during a cleaning interaction [47,48]. Flinches or jolts are correlates of cheating: both Bshary & Grutter [47] and Soares *et al*. [48] showed that non-parasitized fish flinch in the presence of cleaners more frequently than parasitized fish. This is a standard metric to quantify cheating in field-based cleaning research (e.g. [40,47–49]). For all interactions, we identified client reef fishes to species using Humann & DeLoach [50].

Our data did not conform to a normal distribution. Comparative cleaning behaviours (visitation rate h^−1^, clean rate h^−1^, flinch rate min^−1^ and total time spent cleaning) were analysed using non-parametric Mann–Whitney *U*-tests in SPSS v.23 [51]. All data are presented as medians and 25th–75th interquartile ranges (IQR) unless noted otherwise.

### 2.2. Tests of mimicry

To test the hypothesis that *P. yucatanicus* is a cleaner mimic, we make specific assumptions about mimicry in cleaning symbioses. We expect that aggressive (e.g. the fangtooth blenny) and visual protective mimicry (i.e. potential predators mistakenly recognize *P. yucatanicus* as a cleaner and do not prey upon them) are the two most plausible types of mimicry in a cleaner–mimic system. Other types of protective mimicry, such as Batesian, and Müllerian mimicry or reproductive mimicry seem less plausible because true cleaner shrimps are unlikely to be venomous or unpalatable models, or to directly use the cleaner–client relationship to enhance reproduction. Given these assumptions, we established the following expectations for how behavioural interactions between mimetic and client species should proceed under both aggressive and protective mimicry scenarios, recognizing that these may be overly simplified and not mutually exclusive alternatives. (i) Aggressive mimicry, defined here as a species with increased foraging success due to mimetic coloration. Aggressive mimics cheat posing clients and remove live tissue instead of ectoparasites [17–20]. Interactions never result in parasite removal. Under this scenario, we expect client reef fishes to pose at aggressive mimic stations with similar regularity as true cleaners, followed by an immediate cheating event and a rapid termination of the interaction. A large retaliatory response from the client typically follows. (ii) Visually protective mimicry, defined here as a species with reduced predation rates and increased fitness due to convergent coloration with a co-occurring true cleaner species [17]. Under this scenario, we expect client reef fishes to pose in front of mimetic species with similar frequency to true cleaners, but mimetic species should be indifferent towards, and not engage, posing fish.

One caveat to our behavioural expectations of an aggressive mimic is that if client fishes do not rapidly terminate the cleaning interaction following an immediate cheating event, and instead continue to pose and allow repeated cheating events, it may not be possible to distinguish between periodic cheating from a true cleaner species versus cheating from a cleaner mimic through behavioural observations alone. Under this scenario, additional types of data (e.g. gut content analyses) would be needed.

## 3. Results

**Figure 2.**
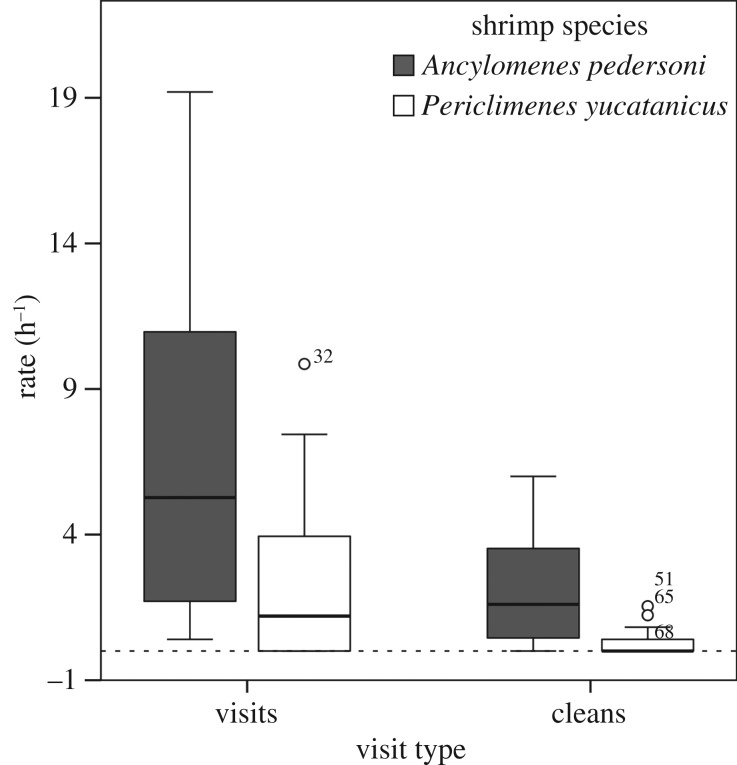
Variation in visitation and cleaning rate (h^−1^) at *P. yucatanicus* and *A. pedersoni* cleaning stations determined through remote video at Curacao, Netherlands Antilles. Data are shown as box plots with median and IQRs for each species and interaction type.

**Figure 3.**
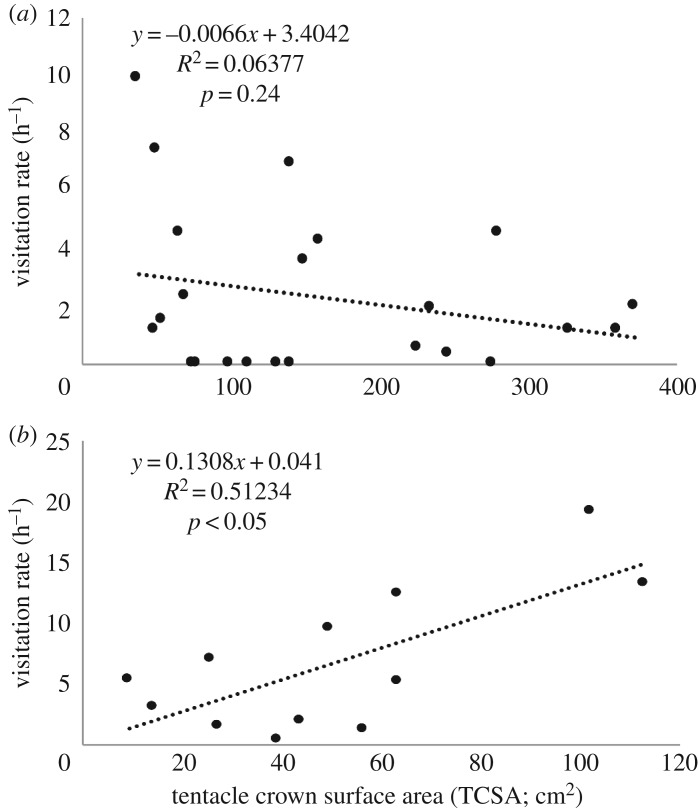
Correlation in client visitation rate (h^−1^) with anemone host size (TCSA) for (*a*) *P. yucatanicus* and (*b*) *A. pedersoni* cleaning stations at Curacao, Netherlands Antilles.

**Figure 4.**
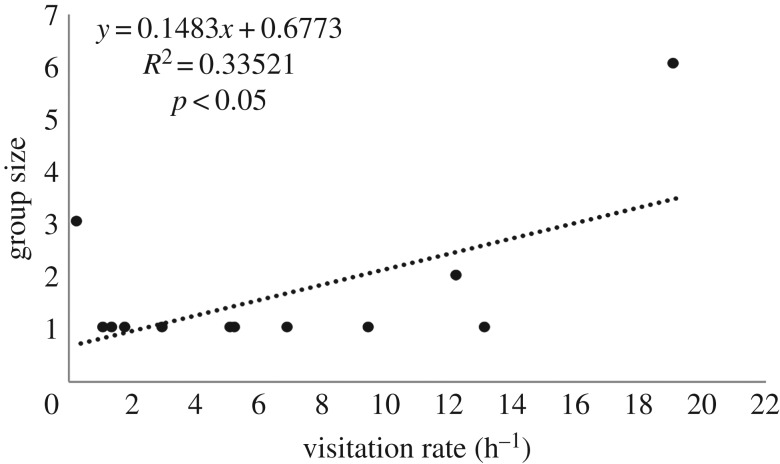
Correlation in client visitation rate (h^−1^) with *A. pedersoni* group size.

*Periclimenes yucatanicus* was slightly more abundant at our study site (1 shrimp/53 m^2^) than *A. pedersoni* (1 shrimp/62 m^2^), and their host anemone *C. gigantea* was significantly larger (141.3 cm^2^ TCSA, IQR: 73–241 cm^2^) than the primary anemone host of *A. pedersoni*, *B. annulata* (46.7 cm^2^ TCSA, IQR: 27–64 cm^2^; *U* = 34.5, *p* < 0.001). In total, we recorded 83 h of video at *P. yucatanicus* (52.48 h) and *A. pedersoni* (30.98 h) cleaning stations. We observed more than 300 visits and 80 cleans at both types of cleaner stations (table 1). Visitation rate (h^−1^) and cleaning rate (h^−1^) were significantly greater at stations hosting *A. pedersoni* (*A. pedersoni*: visitation rate = 5.3 h^−1^, IQR: 1.6–11.6 h^−1^; cleaning rate = 1.6 h^−1^, IQR = 0.4–3.7 h^−1^; *P. yucatanicus*: visitation rate = 1.2 h^−1^, IQR: 0.0–4.2 h^−1^; cleaning rate = 0.0 h^−1^, IQR = 0.0–0.4 h^−1^; visitation rate: Mann–Whitney *U*-test, *U* = 62.5, *p* < 0.01; cleaning rate: *U* = 38.5, *p* < 0.0001; figure 2). There was no significant correlation between anemone size and visitation rate to *P. yucatanicus* cleaning stations (*r*^2^ = 0.06; *p* = 0.24; figure 3*a*), but there was a significant positive correlation between anemone host size and visitation rate to *A. pedersoni* cleaning stations (*r*^2^ = 0.51, *p* < 0.01; figure 3*b*). Additionally, we recovered a significant positive correlation between cleaner group size and visitation rate in *A. pedersoni* (*r*^2^ = 0.34, *p* < 0.05; figure 4). While these trends for *A. pedersoni* were statistically significant, we suggest caution in over-interpreting these trends as sample sizes were small (*n* = 12 stations). Cleaning interactions were recorded at fewer than half (7/23) of the stations containing *P*. *yucatanicus* and at all but one (11/12) station hosting *A. pedersoni* (*χ*^2^ = 3.42, *p* = 0.06). Interaction (clean) length and cumulative time (s) spent cleaning were also significantly greater at stations hosting *A. pedersoni* (table 1; interaction length: *U* = 285, *p* < 0.0001; cumulative cleaning time: *U* = 30.5, *p* < 0.0001). Of the 19 total cleans observed at stations hosting *P. yucatanicus*, 50% were shorter than 10 s in total duration.

**Table 1.** Summary statistics of sample sizes and cleaning data from both *P. yucatanicus* and *A. pedersoni* cleaning stations recorded in Curacao, Netherlands Antilles. *n*, sample sizes; IQR, interquartile ranges.

species	*n*	hours of video	total no. visits	total no. cleans	cumulative clean time (min)	median clean length (IQR) (s)	no. families visited (cleaned)	no. species visited (cleaned)
*Periclimenes yucatanicus*	23	52.48	127	19	6	10 (5–23)	9 (6)	13 (8)
*Ancylomenes pedersoni*	12	30.98	187	66	71.33	32 (15–75)	9 (9)	15 (15)

Both *P. yucatanicus* and *A. pedersoni* were observed cheating client reef fishes. We observed cheating in 16 of 19 interactions at *P. yucatanicus* stations and 49 of 66 interactions at *A. pedersoni* stations. At both types of cleaning stations where cleans were observed (*n* = 7 for *P. yucatanicus* and *n* = 11 for *A. pedersoni*), there was no significant difference in cheating frequency per cleaning interaction (*P. yucatanicus* cheat rate clean^−1^: 0.67 clean^−1^, IQR: 0.0–2.0 clean^−1^; *A. pedersoni* cheat rate clean^−1^: 0.71 clean^−1^, IQR: 0.56–1.08 clean^−1^; *U* = 0.084, *p* = 1.00), or in the frequency of cheating per minute during cleaning interactions (*P. yucatanicus* cheat rate min^−1^: 2.25 min^−1^, IQR: 0.13–5.5 min^−1^; *A. pedersoni* cheat rate min^−1^: 0.86 min^−1^, IQR: 1.04–3.0 min^−1^; *U* = 2.837, *p* = 0.153). At *P. yucatanicus* stations, 7 of 16 flinches occurred in the last 5 s of the interaction, suggesting that the cheating may have terminated the cleaning interaction. At *A. pedersoni* stations, only 9 of 49 flinches occurred during the last 5 s of the interaction. The proportion of flinches that appeared to terminate cleans at *P. yucatanicus* stations was not significantly greater than at *A. pedersoni* stations, but only marginally so (Fisher's exact test, *p* = 0.052).

The numbers of reef fish families and species that visited *P. yucatanicus* and *A. pedersoni* stations were similar (table 1 and tables 2 and 3), but *P. yucatanicus* cleaned a less diverse community of fish (tables 1–3). Species in the family Pomacentrididae (damselfish) were frequent visitors to both cleaner types, yet were infrequently cleaned (tables 2 and 3). Only species in the family Mullidae (goatfish) showed noticeably different patterns of visitation between the two cleaner species, visiting *A. pedersoni* far more often than *P. yucatanicus* (tables 2 and 3). Visits from other families and species were numerically similar, yet per unit time, *P. yucatanicus* stations had reduced visitation rates.

**Table 2.** Diversity and interaction length of client reef fishes observed at *P. yucatanicus* cleaning stations in Curacao, Netherlands Antilles.

family	genus	species	common name	visits	cleans	cumulative clean time (s)	median clean length (s)
Acanthuridae	*Acanthurus*	*bahianus*	ocean surgeonfish	2	0	0	—
		*coeruleus*	blue tang	9	4	215	52.5
Chaetodontidae	*Chaetodon*	*striatus*	banded butterflyfish	2	0	0	—
Haemulidae	*Haemulon*	*plumierii*	white grunt	7	2	2	1
Labridae	*Halichoeres*	*cyanocephalus*	lightning wrasse	1	0	0	—
Mullidae	*Pseudopeneus*	*maculatus*	spotted goatfish	5	1	36	—
Pomacentridae	*Stegastes*	*partitus*	bicolor damselfish	70	3	35	11.7
Scarine labrids (parrotfishes)	*Scarus*	*taeniopterus*	princess parrotfish	15	4	31	6
	*Sparisoma*	*aurofrenatum*	redband parrotfish	2	2	20	10
		*viride*	stoplight parrotfish	3	1	13	—
Serranidae	*Cephalopholis*	*cruentata*	graysby	4	0	0	—
	*Serranus*	*tiginus*	harlequin bass	1	0	0	—
Tetradontidae	*Canthigaster*	*rostrata*	sharpnose puffer	6	2	11	5.5

**Table 3.** Diversity and interaction length of client reef fishes observed at *A. pedersoni* cleaning stations in Curacao, Netherlands Antilles.

family	genus	species	common name	visits	cleans	cumulative clean time (s)	median clean length (s)
Acanthuridae	*Acanthurus*	*bahianus*	ocean surgeonfish	2	1	15	—
		*coeruleus*	blue tang	4	3	108	27
Chaetodontidae	*Chaetodon*	*striatus*	banded butterflyfish	1	1	211	—
Haemulidae	*Haemulon*	*plumierii*	white grunt	4	3	99	33
Labridae	*Halichoeres*	*gamoti*	yellowhead wrasse	3	1	15	—
Mullidae	*Mulloidicthys*	*martinicus*	yellow goatfish	32	21	1936	39
	*Pseudopeneus*	*maculatus*	spotted goatfish	39	23	1255	34
Pomacentridae	*Chromis*	*multilineata*	brown chromis	31	1	18	—
	*Stegastes*	*partitus*	bicolor damselfish	21	3	30	14
Scarine labrids (parrotfishes)	*Scarus*	*taeniopterus*	princess parrotfish	7	2	76	38
	*Sparisoma*	*aurofrenatum*	redband parrotfish	5	1	17	—
		*viride*	stoplight parrotfish	7	1	37	—
Serranidae	*Cephalopholis*	*cruentata*	graysby	3	2	356	178
	*Serranus*	*tiginus*	harlequin bass	7	1	10	—
Tetradontidae	*Canthigaster*	*rostrata*	sharpnose puffer	21	2	97	48.5

## 4. Discussion

We provide the first field-based evidence that reef fish in the tropical western Atlantic visit *P. yucatanicus* with some regularity, and that in combination with its unambiguous signalling (electronic supplementary material, video S1), this species engages in true symbiotic cleaning interactions with reef fishes. Our video observations demonstrate that *P. yucatanicus* wave their long white antennae, signalling to client reef fishes that they are available to clean (electronic supplementary material, video S1). This behavioural signal is the same signal used by *A. pedersoni*, is akin to other overt signalling behaviours that true cleaner shrimp use to engage client reef fish (e.g. [5,25,28,52]) and meets the newly revised definition of a cleaning symbiosis proposed by Vaughan *et al*. [11], which requires communicative behaviours for both cleaner and client. Combined with reciprocal poses from clients and engagement in cleaning activity, our data confirm the designation of *P. yucatanicus* as a true cleaner shrimp species. However, we only deployed cameras during mid-morning and only for a limited period of time, and so we lack the temporal context required to determine whether *P. yucatanicus* is a dedicated or facultative cleaner shrimp. *Ancylomenes pedersoni*, a dedicated cleaner, shows no temporal patterns of cleaning services throughout the day in Honduras, receiving regular visits and engaging in cleaning bouts from dawn to dusk [25]. Other dedicated cleaners (e.g. gobies) show increased cleaning activity at dawn, coinciding with gnathiid isopod abundance (e.g. [53,54]), one of their preferred parasitic food sources [44]. It is unclear at this point how cleaning varies throughout the day for *P. yucatanicus*, and it is certainly possible that its cleaning behaviour may be more temporally restricted. Nocturnal activity should also not be discounted as cleaner shrimp have been demonstrated to forage and clean at night [55,56]. To date, no nocturnal cleaning activity has been documented or observed in the Caribbean for cleaner gobies, juvenile wrasse or *A. pedersoni* [27].

Previous work has shown considerable overlap in clientele between *A. pedersoni* and cleaner gobies in the Caribbean [25]. We demonstrate similar overlap in clientele between *A. pedersoni* and *P. yucatanicus*. Nine families of fish had members who visited both cleaner species at our study site in Curacao, in contrast to more than 20 families whose members have been documented visiting *A. pedersoni* stations throughout its range [25,28–30]. Although the relatively low client diversity here does limit our ability to discern the extent of the community of western Atlantic reef fish that visit *P. yucatanicus* stations, cleaning rates observed in *A. pedersoni* largely mirror those recovered in Honduras [25] and US Virgin Islands [28]. Thus, the rate at which client reef fish visit *A. pedersoni* appears to be independent of region, reef site and local reef fish diversity, giving us confidence that our video data set accurately captures the scope of the usage of cleaner shrimp stations in Curacao. A follow-up study in a higher diversity setting would illuminate whether any client families specialize on specific shrimp species, but to date no client families have been documented as cleaner-shrimp specialists. If *P. yucatanicus* service the same community of reef fishes as *A. pedersoni* and cleaner gobies, as our data suggest they may, understanding how these (apparently) ecologically redundant cleaner species co-occur, compete for clients, and partition resources will be an important avenue for future research. One possibility is that different cleaners specialize on different parasites. Gobies clean more actively during dawn and show reduced cleaning activity as gnathiid isopod abundance naturally decreases throughout the day [53,54,57,58]; by contrast, *A. pedersoni* cleans regularly from dawn to dusk, which may suggest this species targets parasites that are more permanently attached (e.g. flatworms) [25]. More systematic investigation is needed into the ecological role of *P. yucatanicus*, as well as all co-occurring cleaner species, to determine whether specialization allows for overlapping clientele, or whether there are sufficient resources (i.e. parasite loads) to allow for such redundancy in service provisioning.

While there is similarity in client diversity, the differences in visitation and cleaning rates between both species are striking. While some of these differences could be attributed to differences in host use, host size is not an appropriate explanation here. Anemone size has a positive effect on cleaning activity for *A. pedersoni* hosted by *B. annulata*, but because *C. gigantea* is the same size or larger than *B. annulata*, host size is not a general explanation for the differences in visitation or cleaning rate between shrimp species. Whether host species identity impacts cleaning activity is unclear and difficult to parse in our data because of the non-random association we see between host anemone and shrimp. However, fish may use sea anemones as visual cues to locate *A. pedersoni* shrimp, so anemone size may matter on some reefs [29]. This may be especially true for *B. annulata* which has thinner and more transparent tentacles than *C. gigantea*, and would explain why we observed a significant increase in fish cleaning rate with body size in *B. annulata* but not *C. gigantea*.

Because we find regular client visitations to *P. yucatanicus* stations that result in cleaning bouts between shrimp and reef fish, we find no compelling evidence that *P. yucatanicus* is a cleaner mimic. The regularity of visitation to *P. yucatanicus* stations is much greater than we would expect given the species’ apparent apprehension to clean (electronic supplementary material, video S1). In a typical cleaner system, high-quality service provisioning by the cleaner results in repeat visits by the client (e.g. [59])*.* However, visitation to *P. yucatanicus* stations was not rare, and the large discrepancy between visitation and cleaning frequency is somewhat consistent with the behavioural expectations we established surrounding visual protective mimicry. In our view, however, the physical engagement between *P. yucatanicus* and posing reef fish negates the interpretation that this is a visually protective mimic species. We further reject the hypothesis of *P. yucatanicus* as an aggressive mimic. While the duration of cleaning bouts was generally short some exceeded 1 min in total length, and not all cheating bouts resulted in a termination of the interaction. There is also no indication from our data that *P. yucatanicus* showed a significantly greater propensity to cheat than *A. pedersoni*, as would be predicted if it were an aggressive mimic, and cheating did not occur during every interaction. Instead, we demonstrate that reef fishes in the Caribbean appear to tolerate regular cheating occurrences by both species. Our data set is the first to quantify the frequency with which cheating occurs in a cleaner shrimp in the tropical western Atlantic. While cheating occurs frequently in both *P. yucatanicus* and *A. pedersoni*, no aggressive responses were observed by the clients towards the cleaners beyond jolting, and not all cheating resulted in the termination of the interaction (electronic supplementary material, video S2). Our findings are consistent with previous studies of cleaner gobies (e.g. [60]) and previous observations of *A. pedersoni* [28] that the cleaning symbioses in the Caribbean may be a system without punishment.

In conclusion, while *P. yucatanicus* appears to be a true cleaner shrimp, the effectiveness of this species as a cleaner and its ecological role (dedicated or facultative cleaner) remains in question. Less than 15% of all client visits resulted in an actual cleaning interaction, and the brevity of these cleans (table 1), in comparison to those performed by *A. pedersoni* on the same reef, probably resulted in minimal parasite removal. These observations and the findings by McCammon *et al*. [4] that *P. yucatanicus* did not significantly reduce monogenean parasite loads on surgeonfish in a semi-natural setting paint a clearer picture of the ecological importance of *P. yucatanicus*. Additional study will be necessary to discern whether *P. yucatanicus* specializes on different ectoparasites than *A. pedersoni* or whether it varies in its cleaning activities across day or season, but we are sceptical that this species plays a role in ecosystem health that equals that of *A. pedersoni*.

## Supplementary Material

Table S1

## Data accessibility

The data set supporting this article is provided as the electronic supplementary material.
